# Supplementary material for: A Likelihood Approach for Real-Time Calibration of Stochastic Compartmental Epidemic Models
Source: PLoS Comput Biol. 2017 Jan 17;13(1):e1005257. doi: 10.1371/journal.pcbi.1005257 (PMC5240920; doi:10.1371/journal.pcbi.1005257)
Supplement: S1 File — (TAR.GZ) [file pcbi.1005257.s014.tar.gz › HSPH_Online-SI-Revision/Additions/MSS11/n100-extreme_noisy/n100-extreme_noisy_NEW2_table0.pdf]

1	newly 3 weeks spec	7368.61	5818.49	{5384., 6921.}	21.069	0.105099	newly 3 weeks cumul	27409.9	23453.3	{22424., 25986.}	14.4348	0.068506	newly 5 weeks spec	4368.49	2863.4	{2442., 3804.}	34.8815	0.19482	newly 5 weeks cumul	37692.1	30447.5	{28648., 34896.}	19.2206	0.094389	newly 54 weeks cumul	51622.1	36404.9	{33249., 43680.}	29.478	0.156027
2	newly 3 weeks spec	5965.	5960.47	{5849., 6081.}	1.1948	0.00518995	newly 3 weeks cumul	20994.	21065.3	{20867., 21258.}	0.616319	0.00266788	newly 5 weeks spec	3796.	3865.13	{3766., 3978.}	2.23894	0.0095814	newly 5 weeks cumul	29744.	29797.9	{29527., 30049.}	0.571846	0.0024791	newly 50 weeks cumul	42279.	42206.6	{41841., 42501.}	0.458076	0.00199335
3	newly 3 weeks spec	6513.	6258.71	{6149., 6368.}	3.90435	0.0173417	newly 3 weeks cumul	27002.	26741.9	{26520., 26946.}	0.984853	0.0043054	newly 5 weeks spec	3181.	2848.21	{2759., 2929.}	10.4618	0.0480872	newly 5 weeks cumul	34839.	33862.8	{33597., 34112.}	2.80212	0.0123505	newly 55 weeks cumul	41209.	39150.4	{38923., 39357.}	4.99558	0.0222616
4	newly 3 weeks spec	6774.	5385.59	{5269., 5518.}	20.4962	0.0996764	newly 3 weeks cumul	33063.	30043.5	{29734., 30299.}	9.1326	0.0416023	newly 5 weeks spec	2428.	1477.91	{1411., 1541.}	39.1306	0.215847	newly 5 weeks cumul	39680.	34377.9	{34074., 34668.}	13.3622	0.0623032	newly 59 weeks cumul	42886.	35893.6	{35543., 36208.}	16.3047	0.0773109
5	newly 3 weeks spec	8431.	8841.09	{8716., 8964.}	4.86407	0.0205965	newly 3 weeks cumul	31964.	34325.	{34123., 34527.}	7.38634	0.0309436	newly 5 weeks spec	4440.	4278.26	{4194., 4351.}	3.64279	0.0161687	newly 5 weeks cumul	42439.	44854.2	{44553., 45080.}	5.69109	0.024034	newly 56 weeks cumul	51326.	53001.1	{52739., 53249.}	3.26365	0.0139445
6	newly 3 weeks spec	7676.	6990.46	{6669., 7702.}	9.29338	0.0428116	newly 3 weeks cumul	28666.	27578.2	{27099., 28499.}	3.81741	0.0169717	newly 5 weeks spec	4357.	3515.06	{3178., 4554.}	21.0374	0.104696	newly 5 weeks cumul	38952.	36122.8	{35095., 39023.}	7.41505	0.0337648	newly 54 weeks cumul	50195.	43548.9	{41286., 51541.}	14.3022	0.0679153
7	newly 3 weeks spec	7326.	7049.83	{6756., 7409.}	4.34657	0.0194389	newly 3 weeks cumul	27951.	26305.5	{25805., 26913.}	5.88705	0.0264114	newly 5 weeks spec	3953.	4080.08	{3796., 4510.}	5.87756	0.0244757	newly 5 weeks cumul	37123.	35818.6	{34870., 37189.}	3.60863	0.0161038	newly 52 weeks cumul	46099.	46778.7	{44661., 50353.}	4.86353	0.0206345
8	newly 3 weeks spec	7809.	6818.16	{6679., 6957.}	12.6884	0.0589805	newly 3 weeks cumul	28877.	27378.6	{27135., 27651.}	5.18894	0.0231512	newly 5 weeks spec	4404.	3258.8	{3175., 3338.}	26.0036	0.130871	newly 5 weeks cumul	39237.	35427.9	{35140., 35730.}	9.7079	0.0443595	newly 54 weeks cumul	50550.	41590.2	{41222., 41913.}	17.7246	0.0847383
9	newly 3 weeks spec	8491.	6920.45	{6720., 7048.}	18.7122	0.0901215	newly 3 weeks cumul	32010.	27084.9	{26660., 27329.}	15.3862	0.0727037	newly 5 weeks spec	4649.	3305.45	{3162., 3371.}	29.4001	0.151388	newly 5 weeks cumul	43099.	35262.	{34711., 35507.}	18.2686	0.0877758	newly 54 weeks cumul	54004.	41314.3	{40596., 41413.}	23.7454	0.117916
10	newly 3 weeks spec	3970.	6979.24	{6835., 7109.}	75.7995	0.244976	newly 3 weeks cumul	22883.	30968.4	{30760., 31192.}	35.3336	0.1314	newly 5 weeks spec	1101.	2966.39	{2890., 3034.}	169.427	0.430361	newly 5 weeks cumul	26005.	38556.2	{38326., 38763.}	48.2647	0.171034	newly 58 weeks cumul	27241.	43609.	{43405., 43814.}	60.086	0.20435
11	newly 3 weeks spec	6532.	5182.8	{4995., 5310.}	20.6552	0.100877	newly 3 weeks cumul	23257.	20098.8	{19668., 20315.}	13.5796	0.063615	newly 5 weeks spec	3974.	2748.84	{2638., 2893.}	30.8294	0.160669	newly 5 weeks cumul	32341.	26676.7	{26072., 26867.}	17.5144	0.0838866	newly 52 weeks cumul	43101.	32831.3	{32110., 32964.}	23.8271	0.118552
12	newly 3 weeks spec	8024.	7702.15	{7595., 7820.}	4.01109	0.0178083	newly 3 weeks cumul	31244.	28592.7	{28416., 28798.}	8.48595	0.038518	newly 5 weeks spec	4154.	4411.47	{4303., 4500.}	6.19812	0.0260551	newly 5 weeks cumul	41376.	38926.4	{38720., 39118.}	5.92027	0.026507	newly 53 weeks cumul	50863.	50286.1	{50122., 50467.}	1.13418	0.00495557
13	newly 3 weeks spec	7496.	5446.03	{5305., 5565.}	27.3475	0.138938	newly 3 weeks cumul	24982.	20442.2	{20117., 20695.}	18.1722	0.0871656	newly 5 weeks spec	4998.	2984.66	{2856., 3078.}	40.2829	0.22449	newly 5 weeks cumul	36270.	27512.4	{27107., 27803.}	24.1455	0.120135	newly 51 weeks cumul	54152.	34336.5	{33754., 34623.}	36.5924	0.198102
14	newly 3 weeks spec	5024.	8832.51	{8717., 8944.}	75.8063	0.245011	newly 3 weeks cumul	30056.	34334.	{34106., 34587.}	14.2336	0.0577876	newly 5 weeks spec	1153.	4269.33	{4170., 4364.}	270.28	0.568468	newly 5 weeks cumul	33840.	44850.	{44623., 45097.}	32.5354	0.122328	newly 56 weeks cumul	34818.	52990.1	{52792., 53160.}	52.1917	0.182389
15	newly 3 weeks spec	5483.	7681.86	{7571., 7808.}	40.1032	0.146415	newly 3 weeks cumul	26035.	31756.9	{31537., 31966.}	21.9776	0.0862738	newly 5 weeks spec	1932.	3564.15	{3494., 3654.}	84.4798	0.265879	newly 5 weeks cumul	31301.	40638.3	{40408., 40857.}	29.8307	0.113374	newly 56 weeks cumul	33894.	47297.6	{47043., 47506.}	39.5457	0.144713
16	newly 3 weeks spec	6616.	5529.64	{5422., 5659.}	16.4202	0.0779589	newly 3 weeks cumul	30832.	25831.1	{25569., 26119.}	16.22	0.0768744	newly 5 weeks spec	2584.	2092.96	{2008., 2171.}	19.0031	0.0916968	newly 5 weeks cumul	37486.	31374.7	{31081., 31683.}	16.3029	0.0773016	newly 56 weeks cumul	41249.	34434.6	{34044., 34776.}	16.5203	0.0784302
17	newly 3 weeks spec	6952.	6153.35	{6028., 6264.}	11.4881	0.0530369	newly 3 weeks cumul	24739.	22609.9	{22380., 22858.}	8.60641	0.0390996	newly 5 weeks spec	4470.	3530.83	{3443., 3616.}	21.0105	0.102506	newly 5 weeks cumul	34818.	30894.2	{30574., 31160.}	11.2693	0.0519374	newly 51 weeks cumul	48456.	39647.1	{39336., 39947.}	18.1792	0.0871446
18	newly 3 weeks spec	7355.	7344.43	{7175., 7458.}	1.22515	0.00533513	newly 3 weeks cumul	26994.	26473.5	{26182., 26743.}	1.94225	0.00853141	newly 5 weeks spec	4020.	4132.67	{4038., 4222.}	2.9898	0.012749	newly 5 weeks cumul	36463.	36246.9	{35913., 36528.}	0.737515	0.00321884	newly 52 weeks cumul	45100.	45727.7	{45341., 46076.}	1.39188	0.00599543
19	newly 3 weeks spec	8310.	6796.31	{6693., 6904.}	18.2153	0.0873639	newly 3 weeks cumul	31919.	27333.3	{27073., 27546.}	14.3667	0.0673677	newly 5 weeks spec	4512.	3258.37	{3162., 3347.}	27.7844	0.141487	newly 5 weeks cumul	42585.	35363.4	{35048., 35664.}	16.958	0.0807116	newly 54 weeks cumul	52753.	41540.5	{41222., 41827.}	21.2547	0.103783
20	newly 3 weeks spec	6547.	8284.13	{8143., 8433.}	26.5332	0.102171	newly 3 weeks cumul	38691.	37998.7	{37774., 38191.}	1.78941	0.0078458	newly 5 weeks spec	1542.	3115.98	{3038., 3182.}	102.074	0.305441	newly 5 weeks cumul	43499.	46279.	{46058., 46504.}	6.39095	0.026902	newly 59 weeks cumul	44967.	50851.9	{50617., 51062.}	13.0872	0.0534114
21	newly 3 weeks spec	7119.	7897.81	{7638., 8062.}	10.9399	0.0449923	newly 3 weeks cumul	29356.	31207.2	{30907., 31430.}	6.30604	0.0265432	newly 5 weeks spec	3172.	3856.43	{3760., 3997.}	21.5987	0.0842989	newly 5 weeks cumul	37313.	40692.	{40301., 40981.}	9.05585	0.0376223	newly 56 weeks cumul	42719.	48298.8	{48066., 48857.}	13.0616	0.0531969
22	newly 3 weeks spec	6759.	7665.01	{7545., 7762.}	13.4045	0.0545986	newly 3 weeks cumul	29716.	31663.5	{31434., 31877.}	6.55364	0.0275623	newly 5 weeks spec	2827.	3560.84	{3488., 3617.}	25.9583	0.100183	newly 5 weeks cumul	37047.	40533.3	{40304., 40765.}	9.41037	0.0390542	newly 57 weeks cumul	41620.	47269.4	{47010., 47482.}	13.5736	0.0552743
23	newly 3 weeks spec	6270.	6651.7	{6548., 6768.}	6.08772	0.0256289	newly 3 weeks cumul	22584.	23539.8	{23377., 23703.}	4.23198	0.0179945	newly 5 weeks spec	3912.	4326.	{4244., 4413.}	10.5828	0.0436316	newly 5 weeks cumul	31596.	33300.8	{33116., 33464.}	5.39549	0.0228179	newly 50 weeks cumul	43028.	47924.5	{47709., 48088.}	11.3799	0.0468048
24	newly 3 weeks spec	7567.	7101.33	{6984., 7210.}	6.15396	0.0276132	newly 3 weeks cumul	27322.	25135.1	{24965., 25340.}	8.00403	0.0362378	newly 5 weeks spec	4888.	4487.43	{4383., 4576.}	8.19497	0.0371978	newly 5 weeks cumul	38440.	35330.9	{35122., 35559.}	8.08824	0.0366339	newly 50 weeks cumul	52801.	49042.7	{48778., 49276.}	7.11795	0.0320715
25	newly 3 weeks spec	6782.	6926.64	{6819., 7057.}	2.16632	0.00927381	newly 3 weeks cumul	30207.	26669.	{26448., 26865.}	11.7124	0.0541083	newly 5 weeks spec	2743.	3786.92	{3687., 3887.}	38.0576	0.139982	newly 5 weeks cumul	37399.	35632.9	{35398., 35850.}	4.72237	0.0210143	newly 53 weeks cumul	41988.	44985.3	{44770., 45184.}	7.13847	0.0299427
26	newly 3 weeks spec	7069.	6153.55	{6007., 6320.}	12.9502	0.0604109	newly 3 weeks cumul	23874.	22592.7	{22334., 22918.}	5.36693	0.0240427	newly 5 weeks spec	4701.	3554.77	{3432., 3641.}	24.3827	0.121903	newly 5 weeks cumul	34401.	30898.4	{30531., 31240.}	10.1818	0.0467631	newly 51 weeks cumul	50936.	39800.7	{39294., 40102.}	21.8613	0.10743
27	newly 3 weeks spec	6335.	5802.13	{5665., 5961.}	8.41152	0.0382487	newly 3 weeks cumul	22631.	21552.4	{21312., 21943.}	4.7659	0.0212388	newly 5 weeks spec	3937.	3456.41	{3345., 3648.}	12.207	0.0567768	newly 5 weeks cumul	31837.	29532.8	{29142., 30350.}	7.23746	0.0326723	newly 52 weeks cumul	45079.	39318.5	{38662., 41316.}	12.7788	0.0594882
28	newly 3 weeks spec	7243.	5566.56	{5460., 5679.}	23.1457	0.114383	newly 3 weeks cumul	27640.	23901.9	{23644., 24150.}	13.5243	0.0631227	newly 5 weeks spec	4186.	2447.46	{2355., 2519.}	41.5323	0.233223	newly 5 weeks cumul	37414.	30086.7	{29774., 30420.}	19.5845	0.0946762	newly 56 weeks cumul	48001.	34311.9	{34010., 34678.}	28.5183	0.145818
29	newly 3 weeks spec	6769.	6911.99	{6797., 7035.}	2.1727	0.0093016	newly 3 weeks cumul	23489.	24046.	{23761., 24294.}	2.37422	0.0101754	newly 5 weeks spec	4398.	4448.33	{4334., 4538.}	1.7442	0.00746429	newly 5 weeks cumul	33395.	34129.6	{33820., 34402.}	2.20698	0.00946934	newly 50 weeks cumul	46315.	47770.6	{46720., 48114.}	3.15617	0.01345
30	newly 3 weeks spec	8192.	8440.74	{8302., 8564.}	3.03638	0.0129597	newly 3 weeks cumul	34290.	34810.5	{34648., 34992.}	1.51796	0.00653875	newly 5 weeks spec	3775.	3825.61	{3724., 3914.}	1.90755	0.00818764	newly 5 weeks cumul	43617.	44398.7	{44200., 44608.}	1.79212	0.00771155	newly 59 weeks cumul	50418.	51303.9	{51082., 51520.}	1.75705	0.00756212
31	newly 3 weeks spec	7625.	6347.81	{6216., 6469.}	16.75	0.079669	newly 3 weeks cumul	37142.	35716.8	{35419., 35990.}	3.83719	0.0170027	newly 5 weeks spec	2494.	1601.53	{1532., 1678.}	35.7847	0.192618	newly 5 weeks cumul	43977.	40558.8	{40262., 40886.}	7.77268	0.0351482	newly 60 weeks cumul	46875.	42059.	{41745., 42364.}	10.2741	0.0470893
32	newly 3 weeks spec	6240.	8172.05	{8212., 8499.}	33.2277	0.125344	newly 3 weeks cumul	27473.	31354.4	{31547., 32013.}	15.737	0.0639351	newly 5 weeks spec	2353.	4356.88	{4348., 4615.}	88.249	0.274513	newly 5 weeks cumul	33807.	41761.1	{42222., 42810.}	25.3999	0.0988467	newly 57 weeks cumul	37286.	51987.1	{52676., 53686.}	41.5272	0.151195
33	newly 3 weeks spec	8467.	8072.72	{7804., 8243.}	4.74501	0.0211905	newly 3 weeks cumul	28949.	28458.5	{27896., 28758.}	1.92653	0.0084526	newly 5 weeks spec	5209.	4903.22	{4647., 5058.}	5.94049	0.0267972	newly 5 weeks cumul	40750.	39763.5	{38729., 40253.}	2.61234	0.0115206	newly 55 weeks cumul	56254.	53148.	{51050., 53811.}	5.58391	0.025049
34	newly 3 weeks spec	6053.	6509.1	{6338., 6666.}	7.85594	0.0328712	newly 3 weeks cumul	19976.	21467.6	{21106., 21776.}	7.72252	0.0323464	newly 5 weeks spec	4286.	4632.52	{4508., 4763.}	8.49883	0.0354633	newly 5 weeks cumul	29425.	31689.4	{31085., 32166.}	7.97896	0.0333877	newly 50 weeks cumul	43351.	48576.1	{46894., 49392.}	12.4717	0.0511474
35	newly 3 weeks spec	8823.	8578.22	{8208., 8797.}	2.81673	0.0125294	newly 3 weeks cumul	31843.	32436.4	{31639., 32788.}	2.1389	0.00918524	newly 5 weeks spec	5272.	4405.32	{4050., 4565.}	16.4393	0.0783917	newly 5 weeks cumul	44053.	43113.4	{41563., 43615.}	2.13293	0.00942938	newly 55 weeks cumul	57367.	52284.6	{49288., 53076.}	8.85941	0.040437
36	newly 3 weeks spec	6496.	6047.11	{5865., 6210.}	7.49307	0.0342308	newly 3 weeks cumul	23327.	22230.4	{21719., 22579.}	5.0144	0.0225378	newly 5 weeks spec	4189.	3474.74	{3302., 3592.}	17.8458	0.0865447	newly 5 weeks cumul	32804.	30354.9	{29568., 30800.}	7.9091	0.0360991	newly 53 weeks cumul	44915.	38907.6	{37410., 39420.}	14.1548	0.0668174
37	newly 3 weeks spec	6929.	5195.08	{5053., 5318.}	25.0241	0.125167	newly 3 weeks cumul	31730.	27450.7	{27132., 27757.}	13.4868	0.0629367	newly 5 weeks spec	2540.	1545.3	{1477., 1620.}	39.1614	0.216094	newly 5 weeks cumul	38553.	31882.7	{31463., 32248.}	17.3017	0.0825233	newly 58 weeks cumul	42395.	33576.3	{33118., 33978.}	20.8014	0.101303
38	newly 3 weeks spec	5419.	7930.03	{7820., 8043.}	46.3375	0.165329	newly 3 weeks cumul	32641.	35311.6	{35102., 35538.}	8.18183	0.0341494	newly 5 weeks spec	1229.	3179.68	{3098., 3251.}	158.721	0.412761	newly 5 weeks cumul	36650.	43571.2	{43368., 43783.}	18.8844	0.0751217	newly 58 weeks cumul	37768.	48461.3	{48244., 48707.}	28.313	0.108268
39	newly 3 weeks spec	5970.	6618.7	{6470., 6732.}	10.866	0.0447474	newly 3 weeks cumul	21972.	23879.1	{23604., 24166.}	8.67973	0.0361315	newly 5 weeks spec	3152.	3773.54	{3666., 3881.}	19.7189	0.0780645	newly 5 weeks cumul	29579.	32750.2	{32351., 33065.}	10.721	0.0442157	newly 52 weeks cumul	36448.	41583.1	{41149., 41938.}	14.0889	0.0572311
40	newly 3 weeks spec	6214.	5814.06	{5689., 5910.}	6.43611	0.0289378	newly 3 weeks cumul	22734.	21963.4	{21689., 22228.}	3.38959	0.0149945	newly 5 weeks spec	3630.	3266.65	{3180., 3350.}	10.0096	0.0458921	newly 5 weeks cumul	31216.	29648.1	{29295., 29964.}	5.02268	0.0223957	newly 53 weeks cumul	41146.	37626.9	{37273., 37934.}	8.55284	0.0388403
41	newly 3 weeks spec	5376.	5979.49	{5850., 6126.}	11.2256	0.0461402	newly 3 weeks cumul	21206.	22177.5	{21905., 22426.}	4.58125	0.0194357	newly 5 weeks spec	2830.	3386.66	{3299., 3474.}	19.67	0.0778967	newly 5 weeks cumul	27896.	30133.4	{29835., 30405.}	8.02047	0.0334913	newly 54 weeks cumul	33607.	38329.	{37994., 38628.}	14.0507	0.0570886
42	newly 3 weeks spec	4668.	7214.68	{7099., 7338.}	54.5561	0.189046	newly 3 weeks cumul	24350.	30630.6	{30380., 30858.}	25.793	0.0996487	newly 5 weeks spec	1451.	3099.89	{3017., 3204.}	113.638	0.329554	newly 5 weeks cumul	28470.	38547.	{38247., 38793.}	35.3951	0.131596	newly 58 weeks cumul	30111.	43764.7	{43416., 44056.}	45.3444	0.162392
43	newly 3 weeks spec	8069.	7357.12	{7225., 7456.}	8.82241	0.0401619	newly 3 weeks cumul	29202.	26201.2	{26002., 26357.}	10.276	0.0471049	newly 5 weeks spec	4750.	4426.11	{4332., 4506.}	7.05832	0.0317859	newly 5 weeks cumul	40208.	36427.7	{36173., 36612.}	9.40179	0.0429005	newly 51 weeks cumul	53458.	48294.5	{47920., 48472.}	9.80164	0.0447855
44	newly 3 weeks spec	6354.	6780.82	{6658., 6882.}	6.71734	0.0281963	newly 3 weeks cumul	27932.	28811.5	{28640., 28959.}	3.14886	0.0134598	newly 5 weeks spec	2650.	3181.77	{3097., 3264.}	20.0668	0.0793452	newly 5 weeks cumul	34711.	36679.1	{36498., 36846.}	5.66996	0.0239486	newly 57 weeks cumul	39102.	43011.7	{42797., 43186.}	9.99875	0.0413852
45	newly 3 weeks spec	4797.	5529.16	{5404., 5640.}	15.2629	0.0616322	newly 3 weeks cumul	26104.	25843.6	{25586., 26079.}	1.09907	0.00480638	newly 5 weeks spec	1420.	2099.98	{2013., 2175.}	47.8859	0.169724	newly 5 weeks cumul	30202.	31399.2	{31155., 31679.}	3.96394	0.0168719	newly 56 weeks cumul	31840.	34459.4	{34133., 34780.}	8.22688	0.0343243
46	newly 3 weeks spec	8356.	8836.33	{8714., 8940.}	5.74832	0.02425	newly 3 weeks cumul	33903.	34321.1	{34101., 34559.}	1.23535	0.00532583	newly 5 weeks spec	3539.	4261.79	{4177., 4349.}	20.4236	0.080656	newly 5 weeks cumul	42756.	44841.8	{44589., 45121.}	4.87831	0.0206816	newly 56 weeks cumul	48435.	52958.4	{52739., 53209.}	9.33903	0.0387729
47	newly 3 weeks spec	6682.	7856.22	{6666., 8344.}	17.7848	0.0694867	newly 3 weeks cumul	30081.	32611.3	{30546., 33775.}	8.41166	0.0347633	newly 5 weeks spec	2681.	3515.4	{2516., 3986.}	34.0746	0.123887	newly 5 weeks cumul	37114.	41462.	{37239., 43075.}	11.7278	0.0473898	newly 58 weeks cumul	40818.	47720.7	{40758., 50396.}	16.9924	0.0665548
48	newly 3 weeks spec	8702.	7447.63	{7321., 7550.}	14.4147	0.0676373	newly 3 weeks cumul	37680.	34310.	{34048., 34544.}	8.94363	0.040696	newly 5 weeks spec	3585.	2640.16	{2561., 2725.}	26.3554	0.132985	newly 5 weeks cumul	46811.	41469.8	{41229., 41712.}	11.4101	0.0526208	newly 58 weeks cumul	52174.	44942.1	{44668., 45194.}	13.861	0.0648045
49	newly 3 weeks spec	4127.	5037.2	{4945., 5123.}	22.0548	0.086514	newly 3 weeks cumul	27465.	29593.3	{29366., 29788.}	7.74895	0.0324066	newly 5 weeks spec	921.	1393.31	{1342., 1445.}	51.2823	0.179614	newly 5 weeks cumul	30359.	33652.2	{33429., 33861.}	10.8474	0.0447208	newly 60 weeks cumul	31206.	35145.1	{34954., 35372.}	12.623	0.0516225
50	newly 3 weeks spec	6476.	6294.11	{6251., 6552.}	3.39145	0.0164565	newly 3 weeks cumul	23838.	23415.8	{23474., 24088.}	2.34143	0.0109552	newly 5 weeks spec	3992.	3752.8	{3704., 3987.}	6.13828	0.0316858	newly 5 weeks cumul	32964.	32080.	{32352., 32979.}	2.73119	0.0132224	newly 51 weeks cumul	44879.	43053.4	{43877., 44667.}	4.07915	0.0206293
